# Albumin Expands Albumin Reabsorption Capacity in Proximal Tubule Epithelial Cells through a Positive Feedback Loop between AKT and Megalin

**DOI:** 10.3390/ijms23020848

**Published:** 2022-01-13

**Authors:** Rodrigo P. Silva-Aguiar, Diogo B. Peruchetti, Lucas S. Florentino, Christina M. Takiya, María-Paz Marzolo, Wagner B. Dias, Ana Acacia S. Pinheiro, Celso Caruso-Neves

**Affiliations:** 1 Instituto de Biofísica Carlos Chagas Filho, Universidade Federal do Rio de Janeiro, Rio de Janeiro 21941-902, Brazil; silva-aguiar@biof.ufrj.br (R.P.S.-A.); dperuchetti@biof.ufrj.br (D.B.P.); lukazf25@gmail.com (L.S.F.); cmtakiya@gmail.com (C.M.T.); diaswb@biof.ufrj.br (W.B.D.); acacia@biof.ufrj.br (A.A.S.P.); 2 Departamento de Biología Celular y Molecular, Pontificia Universidad Católica de Chile, Santiago 8330163, Chile; mmarzolo@bio.puc.cl; 3 Redes de Pesquisa em Nanotecnologia para Saúde, NanoSaúde/FAPERJ, Rio de Janeiro 21040-900, Brazil; 4 Instituto Nacional de Ciência e Tecnologia em Medicina Regenerativa, INCT-Regenera, Rio de Janeiro 21941-902, Brazil

**Keywords:** albumin endocytosis, AKT, PKB, megalin, proximal tubule, endosome signaling

## Abstract

Renal proximal tubule cells (PTECs) act as urine gatekeepers, constantly and efficiently avoiding urinary protein waste through receptor-mediated endocytosis. Despite its importance, little is known about how this process is modulated in physiologic conditions. Data suggest that the phosphoinositide-3-kinase (PI3K)/protein kinase B (AKT) pathway regulates PTEC protein reabsorption. Here, we worked on the hypothesis that the physiologic albumin concentration and PI3K/AKT pathway form a positive feedback loop to expand endocytic capacity. Using LLC-PK1 cells, a model of PTECs, we showed that the PI3K/AKT pathway is required for megalin recycling and surface expression, affecting albumin uptake. Inhibition of this pathway stalls megalin at EEA1^+^ endosomes. Physiologic albumin concentration (0.01 mg/mL) activated AKT; this depends on megalin-mediated albumin endocytosis and requires previous activation of PI3K/mTORC2. This effect is correlated to the increase in albumin endocytosis, a phenomenon that we refer to as “albumin-induced albumin endocytosis”. Mice treated with L-lysine present decreased albumin endocytosis leading to proteinuria and albuminuria associated with inhibition of AKT activity. Renal cortex explants obtained from control mice treated with MK-2206 decreased albumin uptake and promoted megalin internalization. Our data highlight the mechanism behind the capacity of PTECs to adapt albumin reabsorption to physiologic fluctuations in its filtration, avoiding urinary excretion.

## 1. Introduction

Renal proximal tubule (PT) epithelial cells (PTECs) are highly specialized and adapted toward a reabsorptive phenotype [1]. Despite restrictions to albumin filtration, a significant amount reaches PTs and less than 1% is eliminated in urine [1,2]. Albumin reabsorption in PTECs presents large plasticity [3,4,5], but how PTECs adapt to variations in protein load under physiologic conditions is still an open matter. It is well established that canonical receptor-mediated, clathrin-dependent albumin endocytosis plays a crucial role in PT albumin reabsorption [1]. The albumin receptor is assembled by the association of three proteins: megalin, cubilin, and amnionless [6]. Entry of albumin into PTECs depends on two steps: receptor(s) binding and internalization of the endocytic machinery. Megalin is crucial for the internalization of this complex with albumin targeted to lysosome degradation and megalin recycled to the surface membrane [6,7]. Thus, the mechanisms determining surface megalin expression are a crucial step for total albumin reabsorption.

Megalin (gp330, LRP-2) is a scavenger receptor and a member of the LDL-like receptor family [7,8]. Structurally, megalin possess a large extracellular domain, implicated in ligand association, a single transmembrane domain, and a C-terminal short cytoplasmatic tail [8]. This tail contains two endocytic NPXY motifs, which interact with adaptor proteins, and a NPXY-like motif (NQNY), involved in apical sorting [9]. Other motifs are also present, such as SH3 and PDZ domains, and are involved in receptor–protein interactions, and putative consensus phosphorylation motifs for protein kinase C (PKC) and A (PKA), casein kinase-II, and glycogen synthase kinase-3β (GSK-3β) [8,10]. In this context, it has been shown that megalin is phosphorylated by GSK-3β, which has a negative impact on its recycling and surface expression [10]. On the other hand, it has been shown that megalin mediates the activation of specific signaling pathways such as phosphoinositide-3-kinase (PI3K)/protein kinase B (AKT) [11]. This evidence suggests that megalin could work reciprocally as a sensor and a target of different cellular pathways.

AKT is a central kinase involved in the regulation of several cellular functions [12]. Classically, regulation of the AKT pathway has been described to occur downstream of tyrosine kinases (RTK) and G protein coupled receptor (GPCR) activation. Full activation of AKT depends on phosphorylation of Ser473 and Thr308 residues, mediated by mammalian target of rapamycin complex 2 (mTORC2) and phosphoinositide-dependent kinase 1 (PDK1), respectively [12]. Increasing attention has been given to AKT activation in intracellular platforms, including endosomes, despite its activation at the plasma membrane [12,13]. It has been shown that AKT interacts with megalin at the luminal membrane of PTECs and is modulated by changes in albumin concentration in the lumen of PTECs [11]. This process leads to regulation of the PI3K/AKT pathway [14]. Reciprocally, it has been shown that this pathway also modulates PTECs megalin-mediated albumin endocytosis [15]. However, how the PI3K/AKT pathway is intercorrelated with fluctuations in physiologic albumin concentration in the lumen of PTECs and albumin endocytosis is still an open matter. Based on these observations, we hypothesized that the PI3K/AKT pathway and albumin endocytosis could form a feedback loop to regulate megalin-mediated reabsorption capacity in PTECs.

The aim of the present work was to investigate the intricate network underlying the feedback loop between megalin-mediated albumin endocytosis and the PI3K/AKT pathway and how this process is implicated in modulation of albumin uptake in PTECs by fluctuations in physiologic albumin concentration. Using a combination of in vitro and in vivo approaches, we demonstrated that the PI3K/AKT pathway is required for a high capacity of PTEC albumin endocytosis. AKT regulates megalin trafficking through EEA1+ early endosomes, enhancing its recycling to the plasma membrane and its surface expression. On the other hand, megalin-mediated endocytosis is required by activation of the PI3K/AKT pathway by physiologic albumin concentration. This interplay expands albumin endocytic capacity of PTECs, which we refer to as “albumin-induced albumin endocytosis”.

## 2. Results

### 2.1. PI3K/AKT Pathway Is Required for Maximal Albumin Endocytosis in PTECs

**Figure 1:**
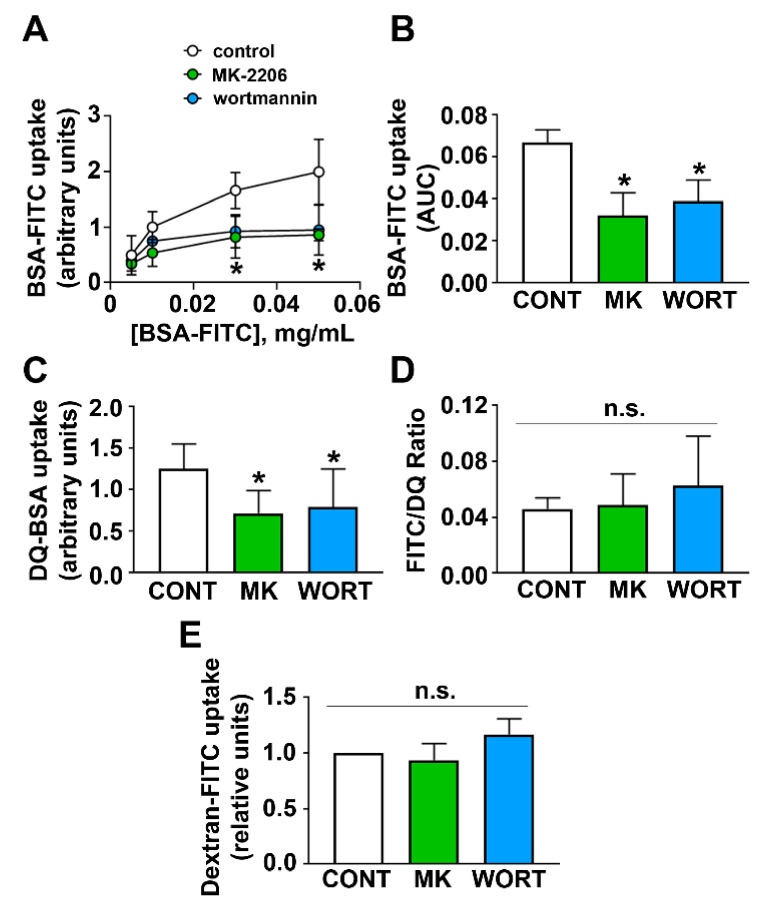
PI3K/AKT inhibition reduces the albumin endocytic capacity of PTECs. (**A**) Dose–response curve of BSA-FITC on BSA-FITC uptake was evaluated in LLC-PK1 cells pre-treated for 1 h with MK-2206 10 µM (MK) or wortmannin 1 µM (WORT) or not (control condition; CONT) (n = 3). (**B**) Area under the curve (AUC) was calculated from the data shown in (**A**). (**C**) DQ-BSA lysosomal degradation (n = 3) and (**D**) dextran-FITC uptake (n = 3) were evaluated under the same experimental conditions described above. (**E**) Ratio between BSA-FITC and DQ-BSA uptake expressed as % of the control. Data are presented as means ± standard deviation. * *p* < 0.05 versus control. One-way ANOVA with Tukey’s multiple comparisons test was used. n.s., no significance.

LLC-PK1 cells were starved overnight before treatment with 10 µM MK-2206 (MK), an AKT inhibitor, or 1 µM wortmannin (WORT), a PI3K inhibitor, for 1 h. FITC-labeled albumin (BSA-FITC) was added at different concentrations, and its uptake was measured. MK-2206 and wortmannin significantly reduced total albumin uptake (Figure 1A,B). Lysosomal degradation, detected by dequenching of DQ-BSA, was similarly reduced by MK-2206 and wortmannin (Figure 1C). The ratio between the inhibition promoted by MK2206 and wortmannin on BSA-FITC uptake and on DQ-BSA lysosomal degradation was not different from control condition, indicating that these effects could be intrinsically overlaid (Figure 1D). The possible effect of the PI3K/AKT pathway on the fluid phase albumin reabsorption was ruled out because dextran uptake was not changed by MK-2206 and wortmannin (Figure 1E). These data indicate that the PI3K/AKT pathway specifically modulates canonical receptor-mediated albumin endocytosis and trafficking to lysosomal degradation.

### 2.2. PI3K/AKT Modulates Intracellular Trafficking of Megalin

**Figure 2:**
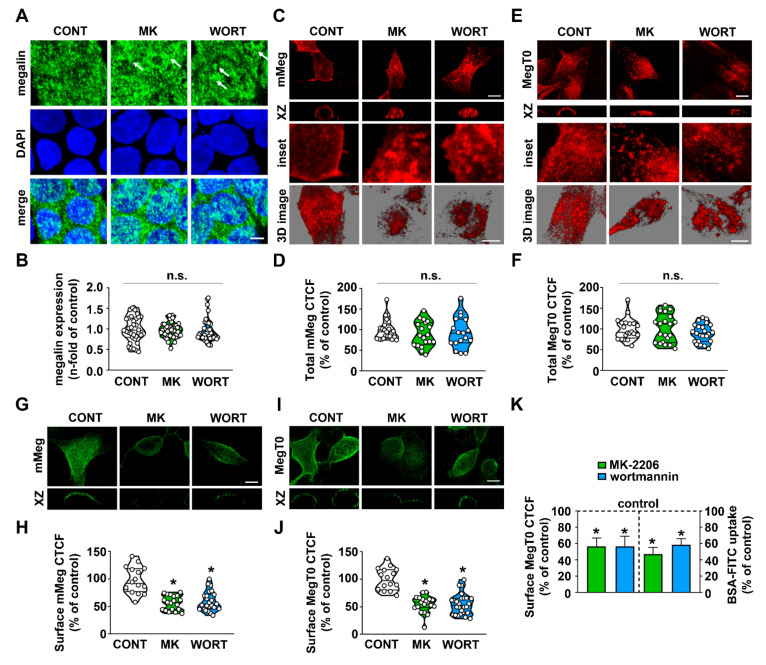
PI3K/AKT inhibition reduces surface megalin expression. (**A**) Representative confocal microscopy images of megalin expression (green) in LLC-PK1 cells. Nuclei were visualized with DAPI staining (blue). Cells were grown in Transwell inserts treated with 10 µM MK-2206 (MK) or 1 µM wortmannin (WORT) for 1 h and compared with control cells (CONT). White arrows indicate areas of megalin signal cluster. Scale bar = 5 μm. (**B**) Quantification of megalin signal intensity per cell was determined (cell number: CONT = 35, MK = 46, WORT = 49; n = 3). (**C**,**E**) Representative confocal microscopy images of immunofluorescence for HA in permeabilized cells (details in the Methods section), expressing mMeg-HA (**C**) and MegT0-HA (**E**), treated with MK or WORT and compared with the CONT. Scale bar = 10 μm. (**D**,**F**) Quantification of mMeg-HA (cell number: CONT = 22, MK = 19, WORT = 17, (**D**)) and MegT0-HA (cell number: CONT = 20, MK = 21, WORT = 23, (**F**)) (n = 3). (**G**,**I**) Representative confocal microscopy images of immunofluorescence for surface HA in non-permeabilized cells (details in the Methods section) expressing mMeg-HA (**G**) and MegT0-HA (**I**). Scale bar = 10 μm. (**H**,**J**) Quantification of surface mMeg-HA (**H**) and MegT0-HA (**J**) signal intensity (cell number: CONT = 22, MK = 19, WORT = 17; n = 3). (**K**) Comparison between surface MegT0 expression (left axis) and BSA-FITC uptake (right axis) in cells treated with MK (green bars) and WORT (blue bars). Results are presented as violin plots. Data are presented as means ± standard deviation. * *p* < 0.05 versus control. One-way ANOVA with Tukey’s multiple comparisons test was used. n.s., no significance.

We then wanted to determine if the modulatory effect of the PI3K/AKT pathway on albumin uptake involves alteration in megalin expression. Total endogenous megalin expression was not changed by the treatment of LLC-PK1 cells with MK-2206 and wortmannin (Figure 2A,B). We used two different megalin constructs (Supplementary Figure S1A) to study the dynamics of megalin trafficking in LLC-PK1 cells: (1) mMeg-HA, which has an HA-epitope at the amino-terminus followed by the fourth-ligand binding domain present in the extracellular domain [10]; and (2) MegT0-HA, which lacks the whole extracellular domain but maintains the HA epitope [7]. Efficient expression of the constructs was confirmed by immunoblotting (Supplementary Figure S1B,C). As observed with endogenous megalin, total mMeg-HA and MegT0-HA expression was not altered by MK-2206 and wortmannin (Figure 2C–F). However, 3D projection confocal microscopy analysis of the luminal cell surface suggested that MK-2206 and wortmannin decreased surface megalin expression (Figure 2C–E). Nonpermeabilized LLC-PK1 cells labeled with anti-HA at 4 °C were used to study surface megalin expression. Both inhibitors decreased surface mMeg-HA and MegT0-HA expression (Figure 2G–J). Furthermore, a similar level of inhibition induced by MK-2206 and wortmannin was observed when surface expression of mMeg-HA or MegT0-HA with the inhibition of albumin uptake were compared (Figure 2K). These findings indicate an association between these phenomena.

**Figure 3:**
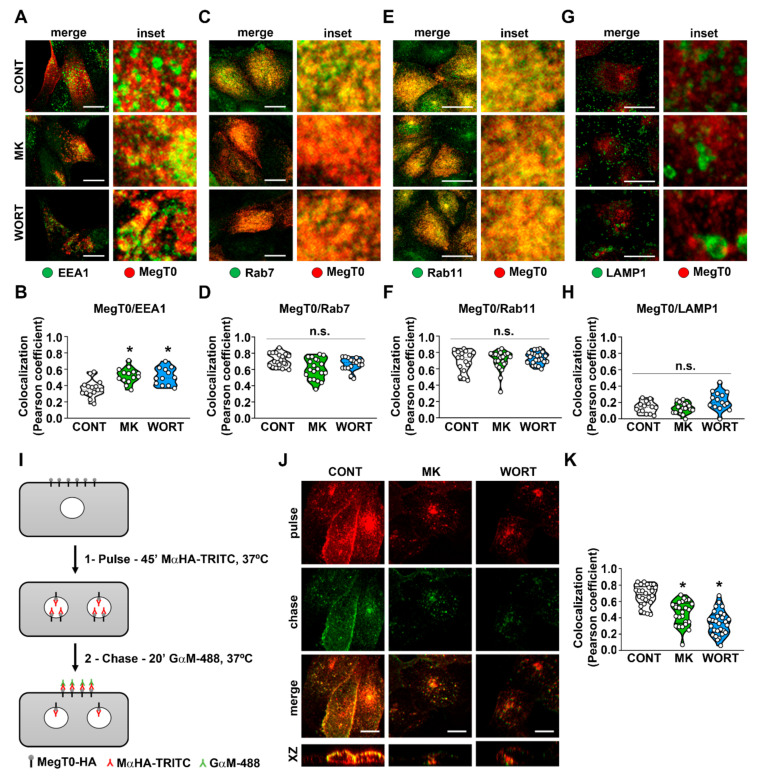
PI3K/AKT inhibition increases megalin presence in early endosomes and reduces megalin cell surface recycling. Representative confocal images showing colocalization of MegT0 and EEA1 ((**A**,**B**) cell number: CONT = 15, MK = 14, WORT = 12; n = 3), scale bar = 20 μm; Rab7 ((**C**,**D**) cell number: CONT = 19, MK = 20, WORT = 19; n = 3), scale bar = 20 μm; Rab11 ((**E**,**F**) cell number: CONT = 17, MK = 22, WORT = 22; n = 3), scale bar = 20 μm; and LAMP1 ((**G**,**H**) cell number: CONT = 14, MK = 19, WORT = 15; n = 3), scale bar = 20 μm, was accessed by immunofluorescence and quantified using Pearson’s coefficient. Results are presented as violin plots. Schematic representation of the pulse-chase approach used to detect MegT0 recycling (**I**). The detailed protocol is described in the Methods section. Representative confocal images of MegT0 recycling in LLC-PK1 cells treated for 1 h with MK or WORT, compared with control cells (CONT) ((**J**,**K**) cell number: CONT = 26, MK = 25, WORT = 28; n = 3), scale bar = 10 μm;. Data are presented as means ± standard deviation. * *p* < 0.05 versus control. One-way ANOVA with Tukey’s multiple comparisons test was used. n.s., no significance.

In the next step, we studied the steady-state distribution of megalin in the endolysosomal system. EEA1, Rab11, Rab7, and LAMP1 were used as markers of early, late, recycling endosomes, and lysosomes, respectively. MK-2206 or wortmannin-treated cells showed an increase in the colocalization of MegT0-HA with EEA1 (Figure 3A,B) but not with Rab7, Rab11, or LAMP1 (Figure 3C–H). The finding that surface luminal megalin expression is decreased and megalin stalls in early endosomes suggests that megalin recycling is regulated by the PI3K/AKT pathway. In this context, megalin recycling was assessed by pulse-chase experiments (Figure 3I–K). Accordingly, megalin recycling to the cell surface was reduced when PTECs were treated with MK-2206 and wortmannin. This result could be associated with a decrease in the total capacity of the albumin endocytosis system (Figure 1).

### 2.3. Megalin-Mediated AKT Activation by Albumin in PTECs

**Figure 4:**
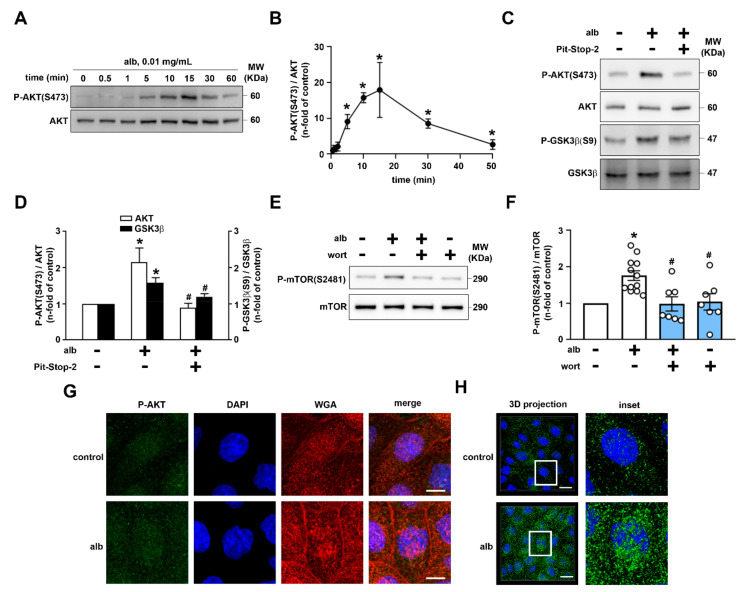
Albumin endocytosis promotes PI3K/AKT pathway activation in LLC-PK1 cells. (**A**,**C**) Representative immunoblotting showing both phosphorylated AKT at serine 473 (P-AKT S473) and total AKT in the cells treated with 0.01 mg/mL albumin. (**A**) Time course of the albumin (Alb) effect and (**B**) densitometric analysis represented as fold of control (n = 3). (**C**) Representative immunoblotting showing P-AKT S473, total AKT, phosphorylated GSK3β at serine 9 (P-GSK3β S9), and total GSK3β of cells treated with 0.01 mg/mL albumin for 15 min in the absence or in the presence of previous pre-treatment with 15 µM Pit-Stop-2 for 15 min (n = 4). (**D**) Densitometric analysis is represented as fold of control. (**E**) Representative immunoblotting showing phosphorylated mTOR complex 2 at serine 2481 (P-mTOR S2481) and total mTOR in the cells treated with 0.01 mg/mL albumin for 15 min in the absence or presence of previous pre-treatment with wortmannin (WORT) for 15 min (n = 7). (**F**) Densitometric analysis is represented as fold of control. (**G**) Representative confocal images of immunofluorescence for P-AKT S473 (green), cell membrane marker (WGA lectin, red), and nuclei (DAPI, blue, n = 3). Scale bar = 10 μm. (**H**) Representative image of 3D projection of a P-AKT S473 positive signal and DAPI are shown. White rectangle represents the inset area. Scale bar = 10 μm. All data are presented as means ± standard deviation. * *p* < 0.05 versus control, ^#^ *p* < 0.05 versus albumin One-way ANOVA with Dunn’s multiple comparisons (AKT, **C**) or Tukey’s multiple comparisons (GSK3β, **C**,**E**) was used.

It was shown previously that physiologic albumin concentration modulates AKT activity, therefore we decided to study the mechanism involved in this process. Pre-incubation of LLC-PK1 cells with 0.01 mg/mL albumin promoted a transient activation of AKT (AKT phosphorylation at serine 473, P-AKT(S473)) with maximal effect observed at 15 min (Figure 4A,B). AKT activation induced by albumin was completely abolished by Pit-Stop-2, an inhibitor of clathrin-mediated endocytosis (Figure 4C,D). Albumin induced GSK-3β phosphorylation at serine 9, an AKT substrate, which promotes its inhibition, and therefore generates a condition in which megalin recycling would be stimulated (10). As a control, we observed that Pit-Stop-2 abolished albumin endocytosis but increased albumin binding (Supplementary Figure S2). Albumin also increased mTORC2 activity, measured by phosphorylation at serine 2481 (S2481), responsible for AKT phosphorylation at S473 (Figure 4E,F). Confocal images revealed that albumin induced AKT phosphorylation (S473), as observed through increased fluorescence intensity, and it was not colocalized with lectin (WGA), a cell surface marker (Figure 4G). In addition, 3D projection of P-AKT(S473) indicates localization of activated AKT in intracellular compartments (Figure 4H). These results suggest that albumin activates AKT by increasing PI3K-required mTORC2 activation, which depends on megalin-mediated endocytosis.

### 2.4. Albumin-Induced Albumin Endocytosis: Role in Albumin Reabsorption in PTECs

**Figure 5:**
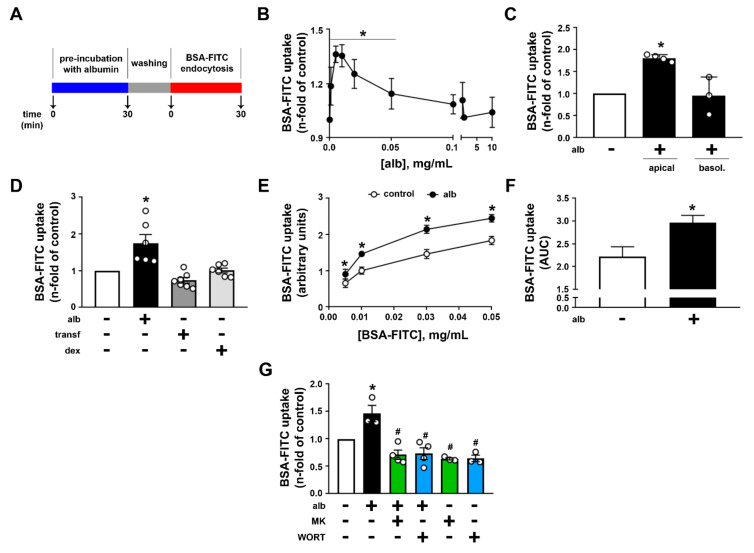
Albumin induces albumin endocytosis through activation of the PI3K/AKT pathway. (**A**) Scheme of the protocol used to study the effect of albumin on BSA-FITC uptake. Cells were pre-incubated with albumin, washed out, and BSA-FITC uptake was measured. (**B**) Dose–response curve of pre-treatment with albumin (alb) for 30 min on BSA-FITC uptake. Albumin concentrations range from 0.001 mg/mL to 10 mg/mL. The graphic represents the fold of control (in the absence of albumin). (**C**) Effect of 0.01 mg/mL albumin added on the apical or basolateral side of LLC-PK1 cells grown in Transwell plates (n = at least 3). (**D**) BSA-FITC uptake was analyzed in LLC-PK1 cells treated with mg/mL albumin, transferrin, or 60 kDa dextran for 30 min. All compounds were used at a concentration of 0.01 mg/mL. Results are expressed as fold of control. (**E**) Dose–response curve of BSA-FITC on BSA-FITC uptake was evaluated in LLC-PK1 cells pre-treated or not with 0.01 mg/mL albumin for 30 min. (**F**) Area under the curve (AUC) of BSA-FITC uptake obtained in (**E**). (**G**) BSA-FITC uptake in LLC-PK1 cells was analyzed after treatment with 0.01 mg/mL albumin for 30 min in the presence or in the absence of MK2206 (MK) and wortmannin (WORT). Data are presented as means ± standard deviation. * *p* < 0.05 versus control, ^#^ *p* < 0.05 versus albumin. One-way ANOVA with Tukey’s multiple comparisons test (**B**–**E**,**G**) or unpaired Student’s t test (**F**) was used.

The possibility that albumin could expand albumin endocytosis was tested. LLC-PK1 cells were exposed to different albumin concentrations (0.001–10.0 mg/mL), washed out, and then albumin-FITC uptake was measured (Figure 5A). This experimental procedure avoids the kinetic effect of changes in albumin concentration on albumin uptake. Albumin pre-incubation increased albumin uptake with maximal effect observed at 0.01 mg/mL (Figure 5B). Further increase in albumin concentration reduced the stimulatory effect, which returned to control conditions at 0.1 mg/mL albumin. The stimulatory effect of albumin pre-incubation on albumin endocytosis was observed only when LLC-PK1 cells were incubated at the luminal side (Figure 5C). Pre-incubation with transferrin and dextran did not change albumin endocytosis, showing a specificity for albumin (Figure 5D). Dose–response experiments on albumin uptake showed that the pre-incubation of the cells with 0.01 mg/mL albumin expanded albumin uptake capacity (Figure 5E,F). We call this phenomenon “albumin-induced albumin endocytosis”, which was completely abolished when the LLC-PK1 cells were pre-treated with wortmannin and MK-2206 (Figure 5G).

**Figure 6:**
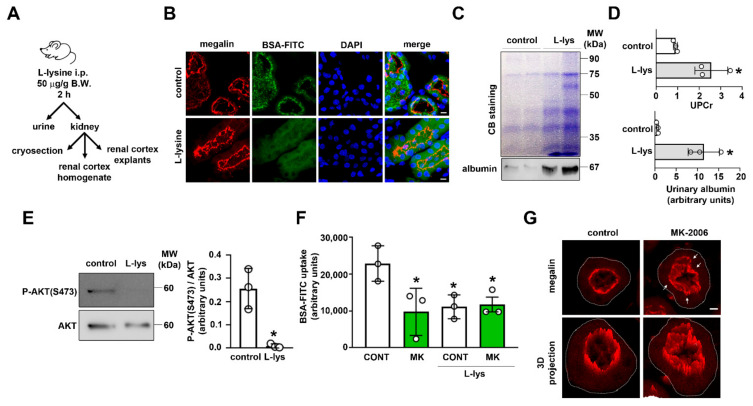
Albumin endocytosis and the PI3K/AKT pathway form a positive feedback loop in an in vivo animal model. (**A**) Scheme of the protocol used to inhibit PTEC protein reabsorption through acute lysine treatment. (**B**) Representative confocal images of BSA-FITC uptake (green), megalin expression (red), and nuclei (DAPI, blue) in proximal tubules from control and L-lysine-treated mice (n = 3). Scale bar = 10 μm. (**C**) Urine samples from control and L-lysine were resolved in SDS-PAGE and stained with Coomassie blue (top panel) or processed for immunoblotting for albumin (bottom panel, n = 3). (**D**) Quantification of the urinary protein/creatinine ratio (UPCr, top graph) or densitometric analysis of urinary albumin excretion (bottom graph) from control and L-lysine-treated mice. (**E**) Representative immunoblotting of phosphorylated AKT (P-AKT S473), and total AKT of renal cortex samples from control and L-lysine-treated mice. Right panel shows densitometric analysis (n = 3). (**F**) BSA-FITC uptake in renal cortex explants from control or L-lysine-treated mice was evaluated in the presence or absence of pre-treatment with 10 μM MK-2206 (MK) for 30 min. (**G**) Representative confocal images of megalin expression (red) in proximal tubules from renal cortex explants of control mice, treated or not with 10 μM MK-2206 for 30 min. A 3D projection of the megalin signal is shown. Basolateral membrane is outlined. Arrows indicate areas of intracellular megalin signal. Scale bar = 5 μm. Data are presented as means ± standard deviation. * *p* < 0.05 versus control. Unpaired Student’s t test (**D**,**E**) or one-way ANOVA with Tukey’s multiple comparisons test (**F**) was used.

To test the possible relevance of these findings in vivo, we used an animal model of acute inhibition of PT megalin-mediated albumin reabsorption obtained by the treatment of BALB/C mice with L-lysine (Figure 6A). It is well accepted that treatment with L-lysine inhibits PT albumin endocytosis due to the inhibition of megalin internalization [16,17]. Here, it was observed that mice treated with L-lysine had lower PTEC albumin endocytosis, visualized by decreased BSA-FITC uptake in PTs (Figure 6B). This effect was associated with low molecular weight proteinuria, albuminuria, and inhibition of AKT activity (Figure 6C–E). The role of the PI3K/AKT pathway was also assessed in ex vivo cortical renal explants obtained from control animals and L-lysine-treated mice. Pre-incubation of control explants with MK-2206 and wortmannin for 30 min inhibited albumin uptake by 50%. On the other hand, cortical renal explants obtained from L-lysine-treated mice presented a lower albumin uptake compared with non-treated mice. MK-2206 did not promote further inhibition (Figure 6F). In addition, MK-2206 induced megalin internalization in control explants obtained from confocal images of frozen sections (Figure 6G, arrows). Taken together, these results corroborate the in vitro findings showing that there is a feedback loop between megalin-mediated albumin endocytosis and AKT activation.

## 3. Discussion

The correlation between the complex endocytic machinery and regulation of albumin reabsorption under physiologic fluctuations of albumin concentration is still an open matter despite its importance [18]. Surface megalin expression is a critical component for albumin uptake and, consequently, in determining the total albumin reabsorption capacity of PTECs [5]. In the present work, we demonstrated that physiologic albumin concentration expands the albumin endocytic capacity of PTECs, a phenomenon we call “albumin-induced albumin endocytosis”. This effect was related to megalin recycling and required activation of an intracellular cascade, the PI3K/AKT pathway, in an intricate feedback loop.

How PTEC endocytosis is modulated under physiologic conditions is a topic of debate. Raghavan et al. [19] showed that an increase in the luminal flow of PTs enhances PTEC albumin endocytosis, which is mediated by fluid shear stress and requires rapid increase of intracellular Ca^2+^. The authors correlated this effect to explain how changes in the glomerular flow rate could be translated to changes in PT function. Additionally, cell stretching mechanical activation was also suggested to enhance protein reabsorption by PT cells [20]. Here, we added a new piece to this puzzle showing that acute changes in luminal albumin concentration under physiologic conditions increased albumin endocytosis. These data indicated that the albumin concentration in the ultrafiltrate could play a relevant role in PT function in addition to changes in the luminal flow. Our observations also present a new perspective on the possible regulatory role of albumin in PT function under physiologic conditions. In agreement with this idea, we showed previously that physiologic albumin concentration increases (Na++K+)-ATPase activity in LLC-PK1 cells, whereas a pathophysiologic concentration inhibits it [21].

Megalin is a long-lived, fast-recycling receptor belonging to the PT endocytic machinery which internalizes several ligands such as albumin [7,8]. Luminal megalin recycling entails its trafficking from early endosomes to the endocytic recycling compartment (ERC), which depends on autosomal recessive hypercholesterolemia (ARH) [22]. Megalin, like polymeric IgA, recycles from the ERC luminal membrane through Rab11+ apical recycling endosomes [8]. Here, it was demonstrated that PI3K/AKT inhibition leads to megalin stall at EEA1+ endosomes but not in Rab11+ and Rab7+ endosomes or in LAMP1+ lysosome. Similar results have been observed in renal proximal tubular dysfunction in Lowe syndrome (LS), in which a stall of megalin is observed in early endosomes associated with a decrease in surface megalin expression and albumin endocytosis [23]. Interestingly, it was proposed that dysregulation of the phosphoinositide metabolism associated with AKT inhibition and clathrin-mediated membrane traffic leads to the neurologic symptoms observed in LS [24].

How AKT promotes this effect is not clear yet. One clue comes from the observation that GSK-3β, an AKT substrate, directly phosphorylates megalin at a PPPSP motif, decreasing its recycling rate in different PTECs lines [10]. Here, we showed that physiologic albumin concentration induces AKT-mediated GSK-3β phosphorylation at the inhibitory site S9. Together these observations support the idea GSK-3β inhibition by AKT is involved in the effect of physiologic albumin concentration on megalin recycling in LLC-PK1 cells. It was shown that megalin anchors AKT at the plasma membrane, and this process is dynamically modulated by the albumin concentration at the luminal side of LLC-PK1 cells, a model of PTECs [11]. Koral et al. [25] showed that endocytic adaptor disabled-2 (Dab2) could be a link between megalin/AKT interaction. Dab2 phosphorylation by AKT is required for PT albumin endocytosis in human kidney proximal tubule clone-8 (HKC-8) cells [26]. The physiologic significance of this process was determined in Dab2 knockout mice, which present albuminuria due to impairment in PT albumin endocytosis [27]. Thus, Dab2, in addition to GSK-3β, could be an effector protein involved in the regulation of albumin endocytosis by PI3K/AKT.

On the other hand, an interesting question is how lower albumin concentration in-creases AKT activity. Previously, it was demonstrated that physiological albumin concentration induces PI3K activation, which in turn increases phosphatidylinositol-3, 4, 5-triphosphate (PIP3) in different membranes [11,14,28]. PIP3 anchors proteins that contain PH domain, such as PDK1 and mTORC2, responsible for AKT activation [12]. In addition, in this condition, AKT is associated to megalin in plasma membrane [11], possibly via Dab2 [25], suggesting that there is a protein assembly that promptly responds to albumin-induced endocytosis, resulting in AKT activation and the occurrence of a positive feedback loop. In agreement with this view, our results show that endocytosis is required for AKT activation induced by albumin, which occurs in endosomal compartments, corresponding to the localization of Dab2 when phosphorylated by AKT [26].

One important question is whether AKT is a constitutive member of the endocytic machinery or a regulator protein in this process. Brunskill et al. [28] showed that the inhibition of PI3K partly abolished albumin endocytosis in OK cells. Previously, our group showed that AKT inhibition induced by high glucose concentration partially abolished megalin-mediated albumin endocytosis [29]. We observed that AKT inhibition did not completely abolish megalin-mediated albumin endocytosis. These data indicate that the PI3K/AKT pathway works as a regulatory component of the endocytic machinery mediated by megalin as a central molecular target for different inputs. In agreement, it was observed that AKT works as a co-adaptor protein required for proper cargo recycling [30].

Spatiotemporal activation of AKT has been suggested as an important step to determine its specific downstream effectors [13]. Endosomal AKT activation was shown to occur due to the activation of different receptors belonging to GPCR and RTK [13,31]. Our results expand this view showing that a scavenger receptor, megalin, induces endosomal activation of AKT. Once activated, AKT increases megalin recycling and albumin endocytosis, which is crucial for maintaining the high capacity of albumin reabsorption by PTECs. In agreement with the role of AKT in albumin reabsorption, it was observed that in a subclinical acute kidney injury animal, activation of AKT enhances PT albumin reabsorption, reduces proteinuria, and ameliorates tubule-interstitial injury [32].

In this work, we demonstrated that the increase in AKT activity induced by albumin involves mTORC2 activation. This result agrees with enhanced albumin endocytosis mediated by mTORC2 activation shown previously [32]. However, it is well known that AKT also regulates mTORC1 activity [12]. In accordance, we also showed that physiological albumin concentrations increase mTORC1 activity in LLC-PK1 cells [14]. Thus, the role of mTORC1 in the feedback loop between megalin-mediated albumin endocytosis and the PI3K/AKT pathway could not be ruled out. Grahammer et al. have shown that mTORC1 regulates proximal tubule protein endocytosis, without changes in megalin expression [33]. Moreover, mTORC1 activity mediates fluid shear stress-induced proximal tubule endocytosis [34]. More results are necessary to confirm this hypothesis. The role of AKT as a central regulator of feedback mechanisms is a hot topic in the literature. It was shown that AKT promotes negative feedback termination of EGFR and insulin signaling, guaranteeing signal fidelity and function specificity [35,36]. In contrast, a positive feedback loop between SREBP and AKT signaling regulates the metabolism of cholesterol in cancer cells [37]. The authors found that the AKT pathway stimulates cholesterol biosynthesis and lipid raft abundance, which was necessary for AKT activation and subsequent proliferation of human melanoma cells. Here, we describe an intricate feedback loop where the AKT pathway is activated by endocytosis of physiologic albumin concentrations, and this activation promotes megalin recycling and surface expression, further expanding albumin endocytic capacity. Together, these results suggest that the AKT pathway fine tunes physiologic and pathophysiologic mechanisms in a context-dependent manner.

**Figure 7:**
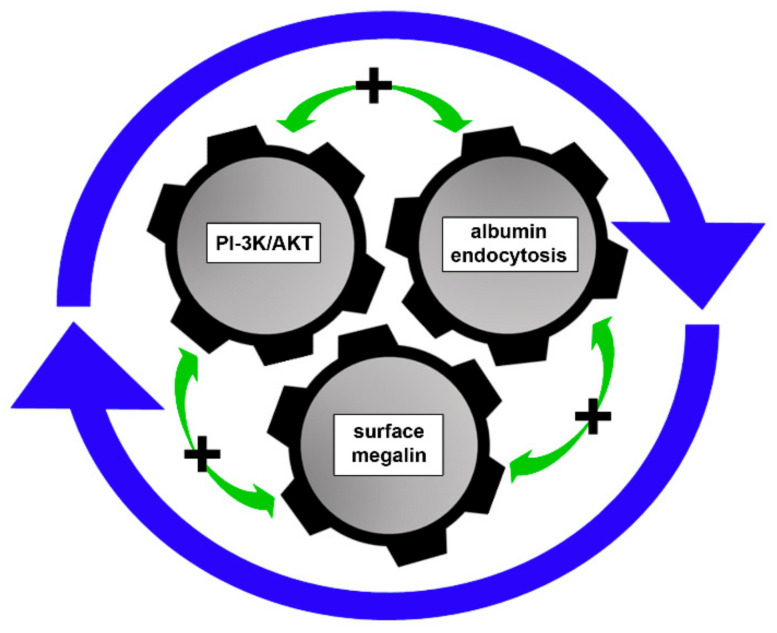
Proposed model. Our results suggest an intricate feedback loop between PI-3K/AKT pathway activation, albumin endocytosis, and surface megalin expression. These 3 components are intrinsically modulated, each being a modulator of the other. This overall process enhances albumin endocytic capacity in PT cells under physiologic conditions.

In summary, we demonstrated the existence of a positive feedback between megalin recycling and activation of the PI3K/AKT pathway. AKT activation by albumin depends on megalin endocytosis, and megalin recycling depends on AKT activation. This process is ultimately associated with megalin surface expression and, consequently, with expansion of albumin uptake in PTECs (Figure 7).

## 4. Materials and Methods

### 4.1. Materials

All reagents were purchased from Sigma-Aldrich (St. Louis, MO, USA), unless otherwise stated. The following antibodies were purchased from Cell Signal Technologies (Danvers, MA, USA): phospho-AKT S473 (#9271), AKT (#9272), phospho-mTOR S2481 (#2974), mTOR (#2972), LAMP1 (Clone D2D11, #9091), EEA1 (#2411), Rab7 (Clone D95F2, #9367), Rab11 (Clone D4F5, #5589), β-actin (Clone 13E5, #4970), anti-rabbit IgG HRP (#7074), and anti-mouse IgG HRP (#7076). The following antibodies were purchased from Abcam (Cambridge, UK): phospho-GSK-3β S9 (ab131097), GSK-3β (Clone 3D10, ab93926), and megalin (ab76969). Mouse anti-HA (HA.11) was purchased from Covance (Princeton, NJ, USA). The following fluorescent antibodies were obtained from Thermo Fisher Scientific (Waltham, MA, USA): anti-rabbit IgG Alexa Fluor Plus 488 (A32731), anti-rabbit IgG Alexa Fluor Plus 594 (A32754), anti-mouse IgG Alexa Fluor Plus 488 (A32723), and anti-mouse IgG Alexa Fluor Plus 594 (A32744). Proteinuria and urinary creatinine colorimetric kits were purchased from Labtest (MG, Brazil). Wortmannin (CAS 19545-26-7) was purchased from Calbiochem (San Diego, CA, USA). MK-2206 (S1078) was purchased from Selleckchem (Houston, TX, USA). Albumin-fluorescein isothiocyanate (FITC) (A9771) was purchased from Sigma-Aldrich. DQ-Albumin Green (D12050) and shRNA for megalin (Ambion) were purchased from Thermo Fisher Scientific.

### 4.2. Cell Culture

LLC-PK1 cells (ATCC, Manassas, VA, USA), a porcine PT cell line, were maintained in low glucose DMEM (Gibco, Thermo Fisher Scientific, Waltham, MA, USA) supplemented with 10% fetal bovine serum (Gibco, Thermo Fisher Scientific, Waltham, MA, USA) and 1% penicillin/streptomycin (Gibco) at 37 °C and 5% CO2. Cells were used between passages 5 and 20. After 2 days of growth, cells were serum starved overnight and different treatments were performed as described in the figure legends. When indicated, cells were seeded in Transwell inserts (Greiner Bio-One, Kremsmünster, Austria) and grown for 5 days. Cells were seeded on coverslips in 24-well plates for microscopy analysis.

### 4.3. Plasmid Transfection

Plasmids containing megalin construct lacking 3 of the 4 extracellular ligand binding domains, mMeg-HA [10], or lacking the whole extracellular domain, MegT0-HA [7], were used. Both constructs possess a hemagglutinin tag, HA, at the extracellular domain, allowing segregation of receptors localized at the surface and in intracellular compartments, as described previously [10]. Briefly, 1 day after plating, LLC-PK1 cells were transfected using Lipofectamine 2000 reagent (Thermo Fischer Scientific) following the manufacturer’s instructions. The next day, transfected cells were selected using 1.0 mg/mL G418 (Sigma-Aldrich, St. Louis, MO, USA). After 9 days of selection, cells were maintained at 0.5 mg/mL G418 and used for the subsequent experiments.

### 4.4. L-Lysine Treatment

All procedures were approved by the institutional ethics committee (CEUA 045-17). Male 8-week-old BALB/C mice, weighing approximately 25 g, were used. The animals were divided into 2 groups of 6 animals in each group: (1) control animals that received intraperitoneal injections of saline (CONT); (2) L-lysine animals (LYS) that received intraperitoneal injections of L-lysine (2 mg/g body weight) as previously described [38]. Control and L-lysine-treated animals were then maintained in metabolic cages for 2 h after the injection for urine collection. Three animals in each group were used for in vivo albumin-FITC endocytosis assay, as described in the next section, and 3 animals were perfused with Ringer’s solution and the kidneys were removed to obtain renal cortex explants by manual dissection using a microtome [39]. Renal cortex explants were equilibrated in low glucose DMEM (Gibco) for 10 min at 37 °C, 5% CO_2_. Renal cortex explants were then washed out and incubated or not with 10 µM MK-2206 (diluted in low glucose DMEM). After incubation for 30 min, renal cortex explants were fixed with paraformaldehyde (PF) 4% for 1 h and processed for immunofluorescence staining.

### 4.5. Albumin Uptake In Vivo

The procedure was conducted as previously described [40]. Briefly, animals were injected intravenously through the tail vein with 5 µg/g body weight of albumin-FITC (Sigma-Aldrich) and 15 min later, the mice were perfused with ice-cold saline, followed by perfusion-fixation with 4% PF. After extraction of the kidneys, samples were incubated overnight in 30% sucrose and then embedded in Tissue Tek OCT (Sakura Finetek, Torrance, CA, USA). Frozen blocks were cut into 10 µm sections on silanized glass slides.

### 4.6. Immunoblotting

Samples from explants or cells were extracted in ice-cold lysis buffer containing 20 mM HEPES, 2 mM EGTA, 1% Triton X-100, phosphatase inhibitor mixture (5 mM Na4P2O7, 50 mM NaF, 5 mM Na3VO4), and protease inhibitor cocktail (Sigma-Aldrich). Samples were pre-clarified by centrifugation at 10,000× *g* for 10 min. The supernatant was collected, and the protein concentration was determined by the Lowry method [41]. Samples were resolved in 9% SDS-PAGE and transferred to polyvinylidene fluoride membranes (Millipore, Burlington, MA, USA). Membranes were probed with specific antibodies overnight at 4 °C, as described in each figure legend, following the manufacturer’s instructions. Detection was performed with ECL Prime (Cytiva, Marlborough, MA, USA), and images were acquired using ImageQuant LAS4000 (GE Healthcare, Chicago, IL, USA).

### 4.7. Immunofluorescence and Confocal Microscopy

#### 4.7.1. In Vitro

Cells were fixed for 15 min with 4% PF and permeabilizated for 15 min in a solution containing 0.05% saponin (diluted in phosphate buffered saline (PBS)) when indicated. Samples were blocked for 30 min in a PBS solution containing 5% bovine serum albumin (BSA) and 0.05% saponin. The samples were then incubated for 1 h with primary and secondary antibodies at room temperature. When indicated, DAPI (Sigma-Aldrich) was added for 5 min, and the cells were mounted with anti-fading mounting medium (Vector Laboratories, Burlingame, CA, USA). For surface expression, cells were placed on ice for 10 min after the treatment time and then incubated with anti-HA antibody on ice for 1 h. Cells were washed twice with ice-cold 1× PBS and then incubated with secondary antibody on ice for 1 h. After 3 washes with ice-cold 1× PBS, cells were fixed with 4% PF for 15 min on ice and the slides were then mounted with mounting medium.

#### 4.7.2. In Vivo

Mouse samples were processed in 2 ways: (1) animals were perfused with 4% PF, followed by overnight incubation in 30% sucrose and OCT embedding. Renal sections of 10 µm were permeabilized with 0.5% PBS-Triton X-100 for 30 min and incubated for 1 h with primary anti-megalin antibody followed by anti-rabbit AF594 Plus; (2) renal cortex explants were maintained in low glucose DMEM with or without indicated treatments for 30 min. The explants were then washed out with PBS, fixed with 4% PF, followed by overnight incubation in 30% sucrose and OCT embedding. Cryosectioning and immunofluorescence were performed as described above. All slides were analyzed in a confocal microscope (Leica SP8, Leica, Wetzlar, Germany) using the LAS X program. Acquisition parameters were the same for all samples in the same experimental group. When indicated, 3D projection was obtained with LAS X. The following analyses were performed using FIJI software. Colocalization analysis was performed using the Coloc2 plugin. Corrected total cell fluorescence, CTCF, was obtained as previously described [42].

### 4.8. MegT0-HA Recycling

Recycling was determined through a pulse-chase approach as previously described [8]. Briefly, starved LLC-PK1 cells stably expressing MegT0-HA plated on coverslips were treated as indicated. Cells were washed out with pre-warmed (37 °C) low glucose DMEM and incubated with 2.5 µg/mL TRITC-mouse anti-HA for 45 min at 37 °C. Cells were then washed out 3 times with ice-cold PBS and recycling was triggered by incubation of pre-warmed (37 °C) low glucose DMEM containing 5.0 µg/mL anti-mouse Alexa Fluor 488 for 20 min. Then, the cells were washed out 3 times with ice-cold PBS, fixed with 4% PF for 15 min, and slides were mounted using mounting medium. Images were obtained on confocal microscope (Leica SP8) using the LAS X program. The analysis was performed with FIJI software version 2.1.0 (NIH, Bethesda, MD, USA). Colocalization analysis was performed with the Coloc2 plugin.

### 4.9. Albumin Uptake and Lysosome Degradation In Vitro

After growth for 2 days, cells were serum starved overnight and treated for at least 30 min with different compounds indicated in the figure legends. After the treatment, cells were washed out with pre-warmed (37 °C) Ringer’s solution and incubated with albumin-FITC for 30 min or DQ-albumin for 60 min. Cells were then washed out with ice-cold Ringer’s solution and lysed in a solution containing 20 mM MOPS-Tris buffer (pH 7.4) and 0.1% Triton X-100. Samples were pre-clarified by centrifugation at 10,000× *g* for 10 min, the supernatant was collected, and cell-associated fluorescence was measured using a fluorimetry SpectraMax M2 (Molecular Devices, San Jose, CA, USA) microplate reader. MK2206 and wortmannin were also maintained during the reaction; pre-incubated albumin was removed from the reaction medium.

### 4.10. Statistical Analysis

All samples are presented as means ± standard deviation. At least 3 independent experiments were performed for each analysis. Colocalization was determined by Pearson’s coefficient, using the Coloc2 plugin with FIJI software version 2.1.0 (NIH, Bethesda, MD, USA). *p* < 0.05 was considered statistically significant. GraphPad Prism version 8 software (GraphPad, San Diego, CA, USA) was used for the statistical analysis. Data distribution was determined by normal QQ plot: normality and lognormality tests. Statistical significance was determined using Student’s t test when comparing 2 groups or one-way ANOVA with appropriate post-tests according to the sample distribution, as described in the figure legends, when comparing 3 or more groups. 

## Data Availability

All data generated or analyzed during this study are present in the paper or the Supplementary Materials.

## Supplementary Materials

The following supporting information can be downloaded at: https://www.mdpi.com/article/10.3390/ijms23020848/s1.
