# Supplementary material for: Albumin Expands Albumin Reabsorption Capacity in Proximal Tubule Epithelial Cells through a Positive Feedback Loop between AKT and Megalin
Source: Int J Mol Sci. 2022 Jan 13;23(2):848. doi: 10.3390/ijms23020848 (PMC8776186; doi:10.3390/ijms23020848)
Supplement: Supplementary file 1 [file ijms-23-00848-s001.zip › ijms-1523838-supplementary.pdf]

Albumin expands albumin reabsorption capacity in proximal tubule epithelial cells through a positive feedback loop between AKT and megalin

Rodrigo P. Silva-Aguiar[1], Diogo B. Peruchetti[1], Lucas S. Florentino[1], Christina M. Takiya[1], Maria P. Marzolo[2], Wagner B. Dias[1], Ana Acacia S. Pinheiro[1,3], Celso Caruso-Neves[1,3,4,*]

[1]Instituto de Biofísica Carlos Chagas Filho, Universidade Federal do Rio de Janeiro, Rio de Janeiro, Brazil

[2]Departamento de Biología Celular y Molecular, Pontificia Universidad Católica, Chile

[3]Redes de Pesquisa em Nanotecnologia para Saúde, NanoSaúde/FAPERJ, Rio de Janeiro, Brazil

[4]Instituto Nacional de Ciência e Tecnologia em Medicina Regenerativa, INCT-Regenera, Rio de Janeiro, Brazil

*Correspondence: Celso Caruso-Neves, Universidade Federal do Rio de Janeiro, CCS, bloco C, sala 34, 21941–902, Rio de Janeiro, RJ, Brazil. Tel.: +55 21 3938 6582; Fax: +55 21 2280 8193; E-mail: caruso@biof.ufrj.br

Supplementary Figure S1 Characterization of ectopic megalin construct expression. (**A**) Scheme of the structures of endogenous megalin, mMeg-HA, and MegT0-HA. Validation of transfection of LLC-PK1 cells with mMeg-HA construct (**B**) or Meg-T0 (**C**) by immunoblotting. Samples were resolved in 9% or 12% SDS-PAGE gels, respectively

Supplementary Figure S2 Effect of Pit-Stop-2 on albumin endocytosis. (**A**) Representative confocal images of BSA-FITC uptake (green) and DAPI (blue) in LLC-PK1 cells treated or not with 15 μM Pit-Stop-2 for 15 min. BSA-FITC surface binding (**B**) or uptake (**C**) was measured under the same experimental conditions. **P* < 0.05 versus control. Unpaired Student's t test (**B, C**) was used

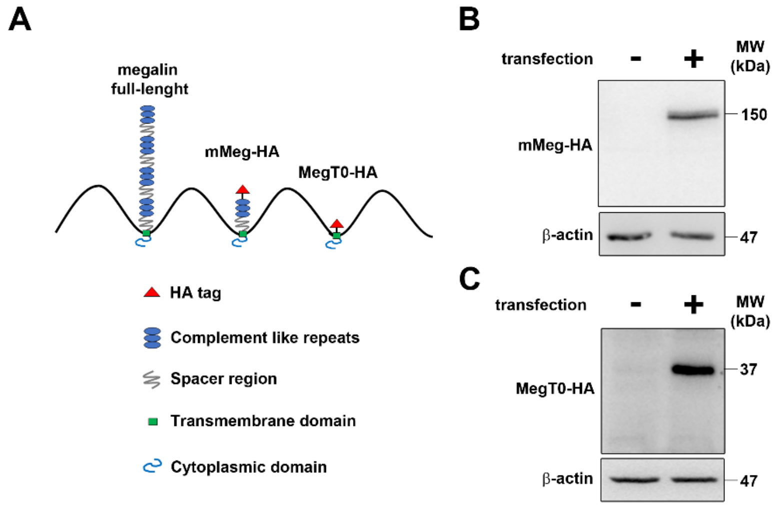

Supplementary Figure S1.

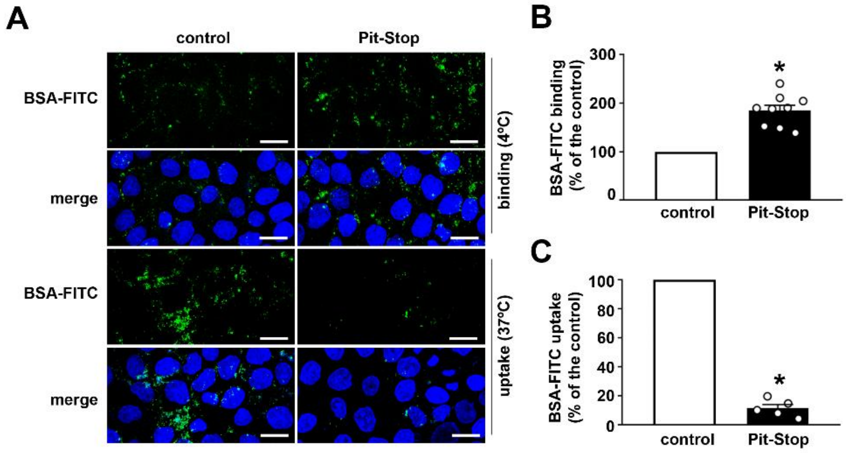

Supplementary Figure S2.
